# In Situ Synthesis of Fluorescent Mesoporous Silica–Carbon Dot Nanohybrids Featuring Folate Receptor-Overexpressing Cancer Cell Targeting and Drug Delivery

**DOI:** 10.1007/s40820-019-0263-3

**Published:** 2019-04-05

**Authors:** Shuai Zhao, Shan Sun, Kai Jiang, Yuhui Wang, Yu Liu, Song Wu, Zhongjun Li, Qinghai Shu, Hengwei Lin

**Affiliations:** 1 School of Material Science and Engineering, Beijing Institute of Technology, Beijing, 100081 People’s Republic of China; 2 Key Laboratory of Graphene Technologies and Applications of Zhejiang Province, Ningbo Institute of Materials Technology & Engineering, Chinese Academy of Sciences, Ningbo, 315201 People’s Republic of China; 3 The Affiliated Luohu Hospital of Shenzhen University, Shenzhen Luohu Hospital Group, Shenzhen, 518001 People’s Republic of China; 4 College of Chemistry and Molecular Engineering, Zhengzhou University, Zhengzhou, 450001 People’s Republic of China

**Keywords:** Mesoporous silica nanoparticles, Carbon dots, Fluorescence imaging, Targeted drug delivery, Chemotherapy

## Abstract

Multifunctional nanocarrier-based theranostics is supposed to overcome some key problems in cancer treatment. In this work, a novel method for the preparation of a fluorescent mesoporous silica–carbon dot nanohybrid was developed. Carbon dots (CDs), from folic acid as the raw material, were prepared in situ and anchored on the surface of amino-modified mesoporous silica nanoparticles (MSNs–NH_2_) via a microwave-assisted solvothermal reaction. The as-prepared nanohybrid (designated MSNs–CDs) not only exhibited strong and stable yellow emission but also preserved the unique features of MSNs (e.g., mesoporous structure, large specific surface area, and good biocompatibility), demonstrating a potential capability for fluorescence imaging-guided drug delivery. More interestingly, the MSNs–CDs nanohybrid was able to selectively target folate receptor-overexpressing cancer cells (e.g., HeLa), indicating that folic acid still retained its function even after undergoing the solvothermal reaction. Benefited by these excellent properties, the fluorescent MSNs–CDs nanohybrid can be employed as a fluorescence-guided nanocarrier for the targeted delivery of anticancer drugs (e.g., doxorubicin), thereby enhancing chemotherapeutic efficacy and reducing side effects. Our studies may provide a facile strategy for the fabrication of multifunctional MSN-based theranostic platforms, which is beneficial in the diagnosis and therapy of cancers in future.

**Electronic supplementary material:**

The online version of this article (10.1007/s40820-019-0263-3) contains supplementary material, which is available to authorized users.

## Highlights

- A one-pot method was developed for the preparation of fluorescent mesoporous silica nanoparticles–carbon dots (MSNs–CDs) nanohybrid.
- The MSNs–CDs nanohybrid showed stable and bright yellow emission, excellent biocompatibility, and specific targeting capability toward folate receptor-overexpressing cancer cells and can be applied as fluorescence imaging-guided drug carriers for effectively delivering anticancer drugs to tumor sites.

**Electronic supplementary material:**

The online version of this article (10.1007/s40820-019-0263-3) contains supplementary material, which is available to authorized users.

## Graphical abstract

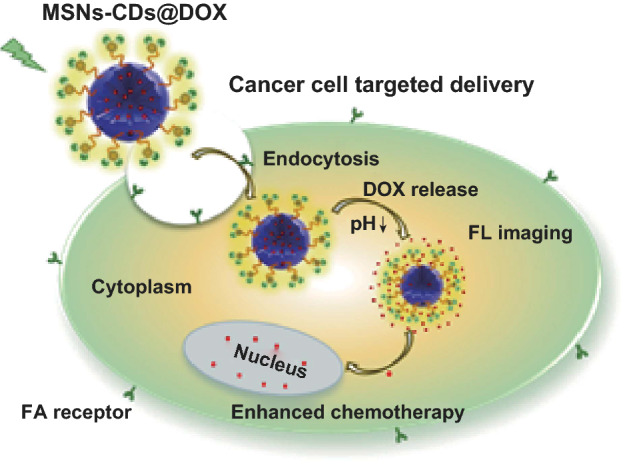

## Introduction

Cancer has become one of the most severe public health problems globally [1]. Currently, chemotherapy is still the major treatment of choice in most cases [2]. However, systemic toxicity, nonspecific interactions, and multidrug resistance are unavoidable, leading to serious side effects [3]. To depress the toxic side effects and simultaneously enhance the therapeutic efficacy, the utilization of targeted drug delivery systems (DDSs) has attracted broad attention [4–7]. In the past few years, various nanomaterials, including micelles [8, 9], liposomes [10, 11], viruses [12, 13], and capsules [14, 15], with good biodegradability and facile functionalization have been designed as DDSs to target cancer cells. However, these nanomaterials tend to suffer from various biochemical attacks and bioerosion, such as enzymatic degradation, on account of their inherent instability in vivo [16–18].

Among various nanomaterial-based DDSs, mesoporous silica nanoparticles (MSNs) have been well demonstrated as excellent carriers for intracellular drug delivery owing to their unique properties, including mesoporous structure, large specific surface area, and pore volume, as well as high biochemical and physicochemical stability, facile surface functionalization, and excellent biocompatibility in particular [19–22]. To further improve their therapeutic efficacy, endowing the DDSs with diagnostic and targeting capabilities has recently been an important issue [23–26]. On the one hand, optical imaging has numerous advantages (e.g., noninvasiveness, high contrast, high sensitivity, and controllable targeting) in comparison with other traditional imaging techniques [27–30]. In recent years, fluorescent MSNs have emerged as an important classification of MSNs, which have been fabricated as multifunctional nanoplatforms and demonstrated great potential in biosensing, bioimaging, and drug delivery [31–33]. In most cases, fluorescent MSNs were prepared by covalently linking organic dyes to the nanoparticles [15, 34]. Organic dyes, however, exhibit inherent limitations of easy photobleaching and complicated synthesis processes [35]. Besides this, semiconductor quantum dots (QDs) and lanthanide-doped upconversion nanoparticles (UCNPs) have also been adopted to synthesize fluorescent MSNs [36–38]. Unfortunately, the highly toxic metal ions in these inorganic phosphor-based fluorescent MSNs are always a great concern and thus limit their further biomedical applications [39]. Therefore, the development of new methods for the facile preparation of fluorescent MSNs that feature high photostability and low toxicity is still highly desirable. On the other hand, in order to increase the local concentration of drugs at the tumor site and reduce side effects, the direct conjugation of MSNs with targeting ligands [5, 40, 41], such as proteins, peptides, and folic acid (FA), is a common approach. However, these methods usually require specialized molecular designs, tedious synthesis steps, and complicated purification procedures [42]. Thus, it is also significant to design novel nanocarriers with facile synthesis procedures and excellent targeting capability.

Carbon dots (CDs), new type of fluorescent carbon-based nanomaterials, have received much attention in recent years [43–46]. Compared with organic dyes, semiconductor QDs, and UCNPs, CDs possess many unique properties, including facile preparation, excellent water solubility, tunable emission, high photostability, and excellent biocompatibility [47–50]. Given these superior properties, CDs have been used to prepare fluorescent MSNs and applied for biosensing, bioimaging, drug delivery, and therapy [14, 51, 52]. In addition, it is well known that folate receptors (FRs) are overexpressed on the surface of certain human cancerous cells, and thus, FA has been frequently employed to conjugate with fluorescent dyes and nanoparticles for selectively imaging and targeting cancer cells [41, 53, 54]. Moreover, we noticed that FA had been used as a carbon source to prepare CDs [55, 56] that showed the capability for selectively targeting FR-overexpressing cancer cells, indicating that FA could retain its function for FR recognition even after undergoing solvothermal carbonization treatment.

Inspired by these above findings, herein, a facile in situ method for the preparation of a fluorescent MSNs–CDs nanohybrid was developed via the solvothermal reaction between FA and amino-functionalized MSNs. Hydrofluoric acid etching of the MSNs confirmed the formation of CDs under the reaction conditions. Not only were the as-prepared fluorescent MSNs–CDs observed to show strong and stable yellow emission, but the unique features of the MSNs (e.g., mesoporous structure and large specific surface area and pore volume) were also retained, indicating the capability of the nanohybrid as an imaging-guided carrier for delivering anticancer drugs (e.g., doxorubicin (DOX)). More specifically, the MSNs–CDs nanohybrid also holds specificity for selectively targeting FR-overexpressing cancer cells (e.g., HeLa cells), implying its potential to enhance the chemotherapeutic efficacy of anticancer drugs and reduce side effects.

## Experimental

### Materials and Instrumentations

All chemicals were purchased from commercial sources and were used without further purification. Tetraethoxysilane (TEOS), ethanol, ammonium chloride (38% aqueous solution), and cetanecyl trimethyl ammonium chloride (CTAC) were obtained from Sinopharm Chemical Reagent Co., Ltd. (Shanghai, China). N-[3-(Trimethoxysilyl)propyl] ethylenediamine (TMS-EDA), triethanolamine (TEA), FA, dimethyl sulfoxide (DMSO), DOX, Hoechst 33258, and 3-(4,5-dimethyl-2-thiazolyl)-2,5-diphenyl-2-H-tetrazolium bromide (MTT) were purchased from Aladdin Chemistry Co., Ltd. (Shanghai, China). Human cervical cancer cells (HeLa), human breast cancer cells (MCF-7), human lung adenocarcinoma cells (A549), and mouse fibroblast cells (L929) were purchased from the cell bank of the Chinese Academy of Sciences (Shanghai Branch). Dulbecco’s modified Eagle’s medium (DMEM) was obtained from Thermo Fisher Scientific Inc., USA. Fetal bovine serum (FBS) was procured from PAN-Seratech (Aidenbach, Germany). Penicillin–streptomycin and trypsin–EDTA were acquired from KeyGEN BioTECH Corp., Ltd. (Jiangsu, China). All aqueous solutions were prepared using deionized water.

The MDS-6G microwave chemical reactor (SMART, Sineo Microwave Chemistry Technology, Shanghai, China) was used to synthesize the MSNs–CDs nanohybrid. Transmission electron microscopy (TEM) was performed to observe the morphology of the nanoparticles, using a Tecnai F20 electron microscope with an acceleration voltage of 200 kV. Specific surface areas were calculated by the Brunauer–Emmett–Teller method, and the pore size distributions were calculated using the Barrett–Joyner–Halenda (BJH) model. Thermogravimetric analysis (TGA) was carried out on a Perkin-Elmer Pyris Diamond TG/DTA instrument, from room temperature to 800 °C at a heating rate of 10 °C min^−1^ under N_2_ atmosphere. Fourier transform infrared (FT-IR) spectra were obtained on a Nicolet 6700 FT-IR spectrometer. Fluorescence spectra were measured on a Hitachi F-4600 spectrophotometer. X-ray photoelectron spectroscopy (XPS) was performed on an Axis Ultra DLD spectrograph with Al/Kα as the source. Hydrodynamic size distributions and zeta potentials were measured through dynamic light scattering (DLS) equipment (Malvern Zetasizer Nano ZS) at room temperature. Small-angle X-ray powder diffraction (XRD) patterns were recorded on a Rigaku D/max-2000 X-ray powder diffractometer (Rigaku, Tokyo, Japan) using Cu/Kα (1.5405 Å) radiation. A microplate reader (iMark 168-1130, Bio-Rad Laboratories, Hercules, CA, USA) was applied for the MTT assay. Cell images were taken with a confocal laser scanning microscope (CLSM, TSCSPS II, Leica, Wetzlar, Germany).

### Synthesis of Amino-Modified MSNs (MSNs–NH_2_)

The MSNs were prepared by following a previously reported procedure [57]. In brief, CTAC (0.5 g) and TEA (0.2 g) were dissolved in 20 mL of distilled water at 95 °C under a 400 rpm stirring rate for 1 h. Then, 1.5 mL of TEOS was added at a speed of 1 mL min^−1^. After continuous stirring for 1 h, the MSNs were collected by centrifugation at 12,000 rpm for 30 min and then washed three times with ethanol and water. To remove the CTAC surfactant, the as-synthesized MSNs were dispersed under ultrasound for 20 min in an ammonium chloride–ethanol solution (4 mg mL^−1^) and then heated to reflux for 12 h. The pure MSNs were then obtained by centrifugation, washed three times with ethanol and water, and finally redispersed in ethanol for further use. The surface modification of amino functional groups on the MSNs was carried out according to our previous report [48]. In brief, 50 mg of MSNs and 5 mL of TMS-EDA were dispersed in 20 mL of ethanol, and then, the mixture was heated and refluxed for 4 h. After cooling to room temperature, the mixture was centrifuged and washed three times with ethanol and water.

### Synthesis of the MSNs–CDs Nanohybrid

The fluorescent MSNs–CDs nanohybrid was prepared through a one-pot solvothermal reaction using FA and MSNs–NH_2_. In brief, 40 mg of FA was dissolved in 40 mL of DMSO and stirred for 1 h, following which 100 mg of MSNs–NH_2_ was added. After ultrasonication for 30 min, the mixture was subjected to microwave heating at 180 °C for 30 min. Then, the reaction mixture was centrifuged at 12,000 rpm for 30 min and the precipitates were collected. After that, the precipitates were washed three times with DMSO and ethanol, respectively, and the MSNs–CDs nanohybrid was finally harvested and redispersed in ethanol for further use.

### DOX Loading Onto and Release from MSNs–CDs

In brief, 4 mg of MSNs–CDs was dispersed in 4 mL of phosphate-buffered solution (PBS) (pH 7.4, 10 mM) in which 2 mg of DOX was dissolved. The mixture was shaken for 24 h at room temperature. The precipitate of MSNs–CDs@DOX was collected by centrifugation and washed three times with PBS buffer to remove free DOX molecules. The DOX loading capacity was calculated from the UV–Vis absorption values. The amounts of DOX released from the MSNs–CDs at two pH values (5.5 and 7.4) were evaluated. In brief, 2 mg of MSNs–CDs@DOX was dispersed in 4 mL of PBS buffer (pH 5.5 and 7.4) and the suspension was shaken at 37 °C. The mixture was refreshed at each run for 2 h, and the supernatant was collected for absorbance measurement to calculate the DOX release efficiency.

### Cell Culture and Cytotoxicity Assay of MSNs–CDs

HeLa, MCF-7, and A549 cells were cultured in DMEM containing 10% FBS and 1% penicillin–streptomycin in a 37 °C incubator with 5% CO_2_. L929 cells were cultured in DMEM containing 15% FBS and 1% penicillin–streptomycin in a 37 °C incubator with 5% CO_2_. To evaluate the biocompatibility of the MSNs–CDs nanohybrid, a cytotoxicity test was carried out, respectively, with HeLa, MCF-7, A549, and L929 cells, using the standard MTT assay. Typically, 100 μL of cells at a density of 1 × 10^5^ cells mL^−1^ was seeded into each well of a 96-well plate and allowed to adhere overnight. Five replicate wells were used for each control and tested concentrations. After culturing in a 5% CO_2_ incubator at 37 °C for 24 h, the culture medium was discarded and the cells were then cultured with 100 μL of DMEM containing various concentrations of MSNs–CDs (0, 20, 40, 60, 80, and 100 μg mL^−1^) for 24 h. After that, 10 μL of MTT (5.0 mg mL^−1^ in PBS) was added into each well and the culture was incubated for another 4 h. Then, the culture medium was removed and 100 μL of DMSO was added to the cells to dissolve the colored formazan. Finally, the absorption intensities of these samples were recorded using a microplate reader at a wavelength of 550 nm.

### Cell-Targeting Study of MSNs–CDs

To evaluate the targeting capability of the MSNs–CDs nanohybrid toward different types of cells, the samples were incubated, respectively, with HeLa, MCF-7, A549, and L929 cells and then visualized by CLSM imaging. For all cell staining experiments, the cells were seeded in glass culture dishes at a density of 5 × 10^4^ cells mL^−1^ and cultured in a 5% CO_2_ incubator at 37 °C for 24 h. The live cells were then incubated with 200 μg mL^−1^ of MSNs–CDs (dispersed in culture medium) for 4 h and thereafter stained with 5.0 μg mL^−1^ of Hoechst 33258 stain for 30 min. After that, the cells were fixed with 4% paraformaldehyde for 30 min. Finally, the samples were rinsed three times with PBS buffer before CLSM imaging. The excitation wavelength was 488 nm, and the fluorescence emission was collected from 500 to 600 nm. To quantitatively evaluate the targeting capability of the MSNs–CDs nanohybrid toward different types of cells, the fluorescence intensities of the samples were also analyzed by flow cytometry.

In order to confirm the effect of FR-mediated cell uptake on the targeting capability of the MSNs–CDs nanohybrid, the cells were treated with excess FA prior to their treatment with MSNs–CDs. HeLa cells were seeded in a glass culture dish at an initial density of 5 × 10^4^ cells mL^−1^ and cultured in a 5% CO_2_ incubator at 37 °C for 24 h. The live cells were then incubated with culture medium containing excess free FA for 2 h. Then, the culture medium was removed and the live cells were incubated with 200 μg mL^−1^ of MSNs–CDs for 4 h, following which they were stained with 5.0 μg mL^−1^ of Hoechst 33258 for 30 min. After that, the cells were fixed with 4% paraformaldehyde for 30 min. Finally, the samples were rinsed three times with PBS buffer before CLSM imaging.

### Therapeutic Efficacy of MSNs–CDs@DOX

To evaluate the therapeutic efficacy of the DOX-loaded MSNs–CDs against cancer cells, free DOX and MSNs–CDs@DOX were subjected to the standard MTT assay, respectively. Typically, 100 μL of HeLa cells at a density of 1 × 10^5^ cells mL^−1^ was seeded into each well of a 96-well plate and allowed to adhere overnight. Five replicate wells were used for each control and tested concentration. After cell culture in a 5% CO_2_ incubator at 37 °C for 24 h, the culture medium was discarded, and the cells were treated with another 100 μL of DMEM containing MSNs–CDs@DOX at a concentration range of 0–100 μg mL^−1^ and equivalent concentration of free DOX for 24 h. At the end of the incubation, 10 μL of MTT (5.0 mg mL^−1^) was added into each well and the culture was incubated for another 4 h. The culture medium was then removed and 100 μL of DMSO was added to the cells dissolve the colored formazan. Finally, the absorption intensities of these samples were recorded using a microplate reader at 550 nm. The therapeutic efficacies of the MSNs–CDs@DOX against MCF-7 and L929 cells were tested in the same way as done for the HeLa cells.

## Results and Discussion

### Design and Preparation of Fluorescent MSNs–CDs Nanohybrid

**Scheme 1:**
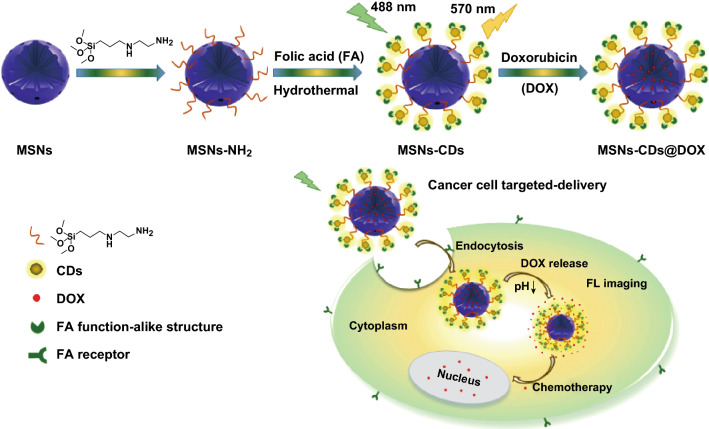
Schematic illustration of the preparation procedures and fluorescence imaging-guided anticancer drug delivery application of the MSNs–CDs nanohybrid

It is known that FA is frequently employed as a targeting ligand to cancer cells [41, 53, 54]. Moreover, FA was recently also used as a carbon resource for the synthesis of CDs, which still retained the capability for targeting FR-overexpressing cancer cells [55, 56]. Inspired by such knowledge, it was surmised that a fluorescent MSNs–CDs nanohybrid with the capability to target FR-overexpressing cancer cells might be achievable via the in situ carbonization of FA on the surface of MSNs. The design and preparation of the MSNs–CDs nanohybrid are shown in Scheme 1. First, MSNs were selected owing to their superior biocompatibility and promising anticancer drug delivery capability. Subsequently, MSNs–NH_2_ were prepared through a common condensation reaction between Si–OH on the MSNs and TMS-EDA. Finally, the MSNs–CDs nanohybrid was prepared via a one-pot solvothermal method, using FA and MSNs–NH_2_ as raw materials. Interestingly, the nanohybrid showed not only stable and bright yellow emission, but also excellent selectivity for targeting FR-overexpressing cancer cells. Besides this, the MSNs–CDs nanohybrid could be efficiently loaded with anticancer drugs (e.g., DOX) and potentially applied for cancer chemotherapy. It is worth mentioning that this work developed a very facile strategy to prepare cancer cell-targetable fluorescent MSNs (i.e., only centrifugation and washing steps being required), which may provide a reference for the highly efficient design and preparation of functional MSNs and implement applications in cancer treatment.

### Characterization of the MSNs, MSNs–NH_2_, and MSNs–CDs

**Fig. 1:**
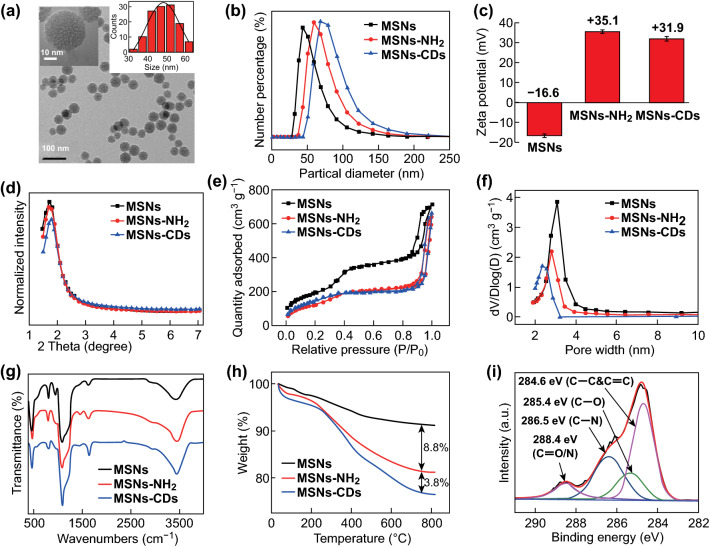
**a** TEM image of the MSNs–CDs nanohybrid (inset: high-resolution image and size distribution of MSNs–CDs). **b** Hydrodynamic diameter distributions, **c** zeta potentials, **d** small-angle XRD patterns, **e** N_2_ adsorption–desorption isotherms, **f** pore size distribution curves, **g** FT-IR spectra, and **h** TGA curves of MSNs, MSNs–NH_2_, and MSNs–CDs. **i** High-resolution C 1*s* XPS spectrum of the MSNs–CDs nanohybrid and fittings

First, the structure and composition of these MSN-based materials were characterized. As shown in Figs. 1a and S1, the TEM images of the MSNs, MSNs–NH_2_, and MSNs–CDs showed them to have a similar spherical mesoporous morphology and diameter distribution (~ 45 nm), indicating that the structure of the MSNs was not damaged after the amino modification and solvothermal treatments. DLS measurements revealed that the hydrodynamic diameters increased from about 50 nm for the MSNs to ~ 63 nm for the MSNs–NH_2_, and to ~ 75 nm for the MSNs–CDs (Fig. 1b), which were larger than those observed by TEM owing to the existence of a hydration layer on the nanoparticles. The zeta potentials of the MSNs, MSNs–NH_2_, and MSNs–CDs were measured to be − 16.6, + 35.1, and + 31.9 mV, respectively, demonstrating the successfully modified amino functional groups on the MSNs and further anchored CDs on the MSNs–NH_2_ (Fig. 1c). The small-angle XRD patterns displayed obvious diffraction peaks corresponding to the (100) panel (Fig. 1d), indicating the well-ordered two-dimensional hexagonal structure of these mesoporous materials [40, 58–60]. In addition, the XRD results also demonstrated that the mesoporous structure of the MSNs was retained, even after they had undergone a two-step chemical reaction. Typical N_2_ adsorption–desorption isotherms (Fig. 1e) and BJH pore size distribution plots (Fig. 1f) were measured to further investigate the mesoporous structure. The isotherms exhibited typical type IV curves with an H1 hysteresis loop, which is characteristic of an ordered mesoporous structure [40]. The MSN type was thus determined to be MCM-41 according to these results [20, 40, 58, 60]. The specific surface area, pore volume, and pore diameter were found to gradually decrease from the MSNs to the MSNs–NH_2_, and to the MSNs–CDs (Table S1). Specifically, the surface area, pore volume, and pore diameter of the MSNs–CDs were 502.94 m^2^ g^−1^, 0.88 cm^3^ g^−1^, and 2.20 nm, respectively, which met the requirements for drug loading. The FT-IR spectra of all the MSN-based materials exhibited no obvious differences, probably due to their dominating silica contents (Fig. 1g), making it difficult to determine the successful conjugation of CDs onto the MSNs. Consequently, TGA and XPS measurements were performed. As shown in Fig. 1h, the TGA results showed major weight losses of 8.8% and 12.6% for the MSNs–NH_2_ and MSNs–CDs, respectively. The grafting CDs could thus be confirmed and was calculated to be 3.8 wt% for the MSNs–CDs nanohybrid. Moreover, the composition and functional groups of the MSNs–CDs were examined by XPS (Fig. S2). The major elements in the MSNs–CDs were determined to be C, N, O, and Si. The high-resolution C 1*s* XPS spectrum (Fig. 1i) exhibited the characteristic peaks of C=C/C–C, C–N, C–O, and amide carbonyl (C=O) at 284.6, 285.4, 286.5, and 288.4 eV, respectively, further indicating the formation of CDs that should be anchored to the MSNs through amide bonds. These characterization results not only clearly confirm the successful modification of CDs on the surface of the MSNs, but also demonstrate that the MSNs–CDs nanohybrid still possesses a unique mesoporous structure.

**Fig. 2:**
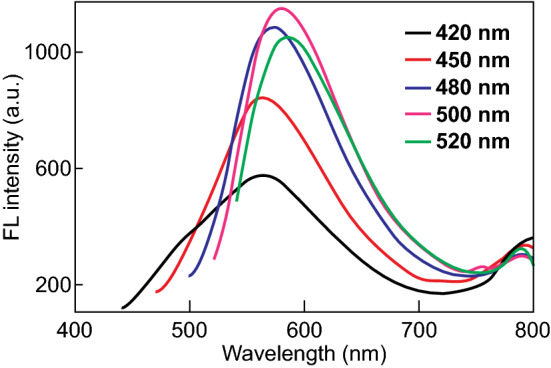
Fluorescence emission spectra of MSNs–CDs under diverse excitation wavelengths

Subsequently, the optical properties of the MSNs–CDs nanohybrid were investigated. Figure S3 shows the excitation and emission spectra of the MSNs–CDs. The optimum excitation and emission wavelengths were 510 and 575 nm, respectively. As shown in Fig. 2, the emission wavelength of the MSNs–CDs only red-shifted slightly with the increase in the excitation wavelength from 420 to 520 nm. The emission was mainly attributed to the carbonization of the FA precursor and eventual formation of the fluorescent CDs during the solvothermal reaction. To verify the formation of the CDs, the MSNs–CDs nanohybrid was treated with hydrofluoric acid to etch the silica framework. As shown in Fig. S4, the TEM image indicated that spherical particles with an average diameter of ~ 4.8 nm were present in the remaining solution. Since the observed particle size of the CDs was apparently larger than the pore diameter of the MSNs–NH_2_ (2.48 nm), we can infer that the CDs were mainly formed and grafted on the outer surface of the MSNs. Furthermore, the photostability of the MSNs–CDs was evaluated. The results displayed the stable fluorescence emission of the MSNs–CDs upon continuous irradiation with UV light (Fig. S5) and nearly constant emission at pH values ranging from 3 to 10 (Fig. S6). Such a highly stable emission property makes this nanohybrid more suitable for bioimaging than the traditional MSNs modified with organic fluorescent dyes.

### Drug Loading and Release Properties of MSNs–CDs

**Fig. 3:**
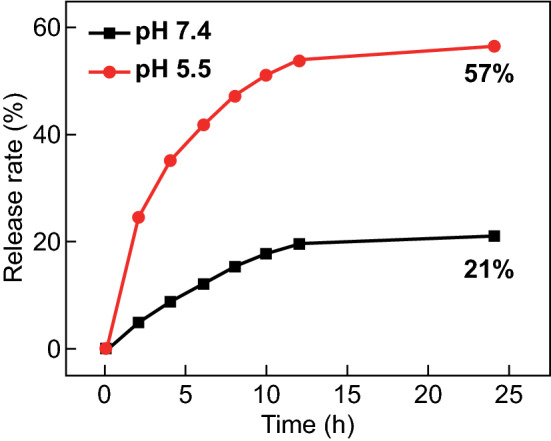
In vitro DOX release curves of DOX-loaded MSNs–CDs in phosphate buffers (10 mM) of pH 7.4 and 5.5 at 37 °C

Owing to the high specific surface area and pore volume of the MSNs–CDs, the nanohybrid is expected to load and deliver drugs for cancer therapy. DOX, a model chemotherapeutic drug, was chosen for such investigation [61]. The DOX loading capacity (in weight) of the nanocarrier was determined to be ~ 250 mg g^−1^ (MSNs–CDs) after an adsorption balance between DOX and the nanohybrid. It is also well known that the tumor microenvironment is faintly acidic, with a pH value of approximately 4.0–7.0 (extracellular 5.7–7.0, intracellular endosomal 5.5–6.0, and lysosomal 4.5–5.0), which is lower than that of the normal physiological condition (pH 7.4) [62]. Therefore, the effective release of DOX from the nanohybrid under an acidic environment is important in cancer therapy. The capability for controllable DOX delivery by the MSNs–CDs at different pH values (pH 5.5 and 7.4) was assessed. As shown in Fig. 3, the cumulative DOX release efficiency was relatively low and was calculated to be 21% after 24 h at pH 7.4. However, a substantial increase in drug release (up to 57%) was observed at pH 5.5, much higher than that in the pH 7.4 medium. The enhanced release efficiency in the acidic solution could be mainly ascribed to the reduced hydrophobic π-π interaction and hydrogen bond interaction between DOX and the MSNs–CDs, which consequently results in the dissociation of DOX from the MSNs–CDs nanohybrid. This pH-dependent drug release behavior could minimize the latent damage to normal cells and enhance the therapeutic efficacy against cancer cells owing to the acidic microenvironment of the tumor and intracellular acidic endosomes and lysosomes [61, 62].

### Cancer Cell-Targeting Capability of MSNs–CDs

**Fig. 4:**
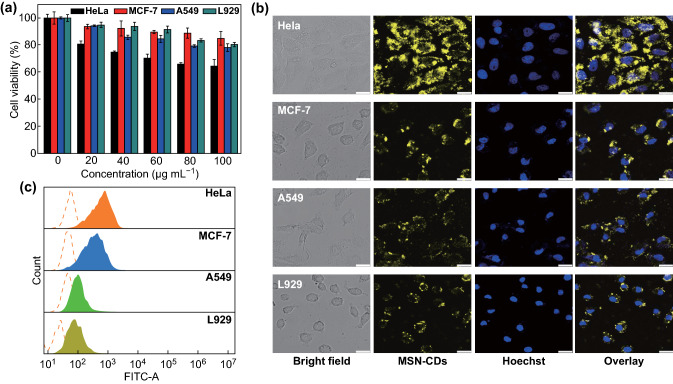
**a** Cytotoxicity of MSNs–CDs at different concentrations (0, 20, 40, 60, 80, and 100 µg mL^−1^) toward four types of cells (HeLa, MCF-7, A549, and L929). **b** CLSM images and **c** flow cytometric analysis results of different cells incubated with 200 μg mL^−1^ of MSNs–CDs for 4 h. Scale bar in **b** is 25 μm

Bestowed with superior optical properties and potential drug delivery capability, the MSNs–CDs nanohybrid was proposed as a fluorescence imaging-guided carrier to deliver anticancer drugs. Meanwhile, FA had been used as a carbon source to prepare CDs with the feature of targeting FR-overexpressing cancer cells [55, 56]. Before discussing the cancer cell-targeting capability of the MSNs–CDs, their biocompatibility was first evaluated by the standard MTT assay. Three types of cancer cells with different levels of FR overexpression (HeLa, MCF-7, and A549) and one kind of normal cell (L929) were chosen as cell models. As shown in Fig. 4a, over 80% of the MCF-7, A549, and L929 cells were viable after being incubated with different concentrations of MSNs–CDs for 24 h. In contrast, the viability of the HeLa cells was relatively low (about 70%), which may mainly be attributed to the overexpression of FR-α on HeLa cells and thus more materials being taken up. (Other factors, such as the survivability of different cell lines to exogenous substances, could also affect the apparent cytotoxicity of the MSN-CDs.)

To demonstrate whether the MSNs–CDs could selectively target FR-overexpressing cancer cells, the above-mentioned four types of cells were, respectively, stained with the nanohybrid. Figure 4b shows the results of confocal fluorescence imaging, where remarkable differences among the four types of cells were observed. Specifically, the brightest fluorescence intensity was observed from the HeLa cells, which was attributed to their overexpression of the FRs and consequently effective surface binding and uptake by the cells via the close rapport between the FA and FRs [41]. The MCF-7 cells showed relatively weaker fluorescence than that of the HeLa cells, which is in good accordance with their medium or low FR expression. The A549 cells, however, are known to be deficient in FR expression and thus exhibited almost no fluorescence [55]. In addition, the normal L929 cells also displayed very weak fluorescence, indicating weakly expressed FRs on this kind of cell. To further confirm these results, quantitative analyses were performed using flow cytometry. The blank groups of the four types of cells showed similar and low fluorescence intensities (represented by the red dotted lines in Fig. 4c). After being, respectively, stained with the MSNs–CDs, the experimental group of HeLa cells showed the highest peak shift in fluorescence intensity, followed by the experimental groups of MCF-7 cells, L929 cells, and A549 cells (Fig. 4c). The flow cytometry data were most consistent with the confocal cellular imaging results. These studies evidenced that the efficiency of cells for MSNs–CDs uptake is FR expression dependent, that is, the MSNs–CDs were able to selectively target FR-overexpressing cancer cells.

**Fig. 5:**
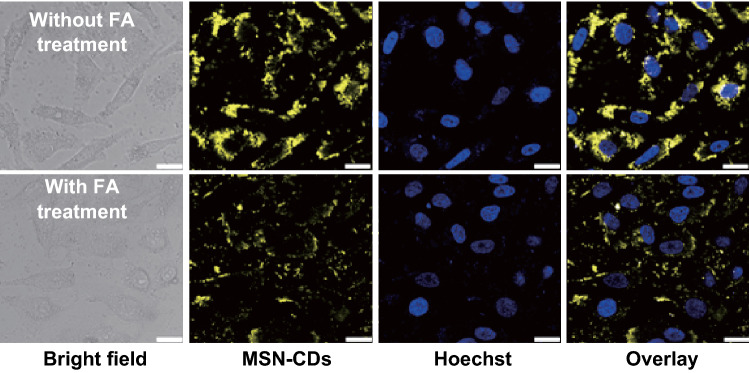
CLSM images of HeLa cells incubated with MSNs–CDs (200 μg mL^−1^) for 4 h. As a comparison, HeLa cells were pretreated for 2 h with excess folic acid (FA) for folate receptor (FR) saturation and then incubated with MSNs–CDs (200 μg mL^−1^) for another 4 h. Scale bar is 25 μm

To clarify the targeting role of the FA function-alike structure of the MSNs–CDs, a competition assay was carried out. The FRs on the surface of HeLa cells were first excessively saturated with FA, followed by treatment with the MSNs–CDs. Compared with the control group (without FA pretreatment), the FA treatment group showed very weak fluorescence (Fig. 5), indicating that the internalization of MSNs–CDs into HeLa cells should be predominantly through an FR-mediated endocytosis manner [56]. This result demonstrates that the MSNs–CDs could be applied for the fluorescence imaging-guided delivery of anticancer drugs to FR-overexpressing cancer cells.

### Cancer Cell Therapeutic Efficacy of DOX-Loaded MSNs–CDs

**Fig. 6:**
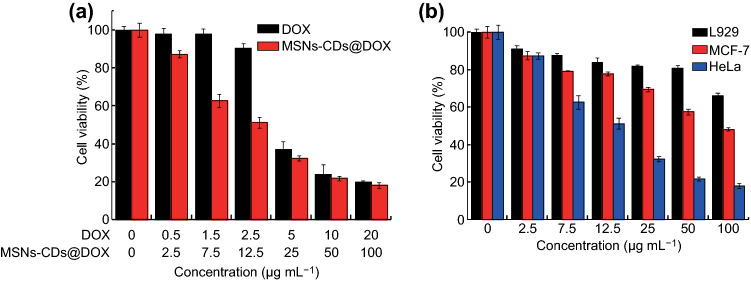
**a** Therapeutic efficacy of MSNs–CDs@DOX and equivalent concentrations of free DOX against HeLa cells at different concentrations. **b** Therapeutic efficacy of MSNs–CDs@DOX against L929, MCF-7, and HeLa cells at different concentrations

Finally, DOX-loaded MSNs–CDs (designated MSNs–CDs@DOX) were preliminarily evaluated for enhanced chemotherapeutic effects against cancer cells in vitro. The therapeutic efficacies of free DOX and MSNs–CDs@DOX against HeLa cells were examined by MTT assay. Figure 6a shows the viability of HeLa cells treated with MSNs–CDs@DOX at concentrations ranging from 0 to 100 μg mL^−1^ or with equivalent concentrations of free DOX. Obviously, the MSNs–CDs@DOX showed higher therapeutic efficacy against HeLa cells than that of the equivalent concentrations of free DOX. In order to clarify that the specific targeting capability of MSNs–CDs toward the FR-overexpressing cancer cells can reduce toxic side effects toward normal tissues, MTT assays of MSNs–CDs@DOX against MCF-7 and L929 cells were also performed. As displayed in Fig. 6b, much higher cell viability was observed for the L929 cells than for the HeLa and MCF-7 cells (particularly for HeLa cells) after their treatment with MSNs–CDs@DOX, clearly indicating the lower side effects of the treatment (i.e., by MSNs–CDs@DOX) toward normal cells or tissues. These results above demonstrate that the MSNs–CDs nanohybrid could be potentially employed as an anticancer drug carrier to enhance the chemotherapeutic efficacy while also reducing side effects.

## Conclusion

In summary, a novel one-pot and in situ method for the preparation of fluorescent MSNs–CDs nanohybrid was developed through the solvothermal treatment of FA and amino-modified MSNs in this study. The nanohybrid not only shows stable and bright yellow emission but also retains the superior features of MSNs. Interestingly, the nanohybrid holds specific targeting capability toward FR-overexpressing cancer cells (e.g., HeLa cells) owing to the FA function-alike structure of the CDs on the MSNs, demonstrating its potential application as a nanocarrier for effectively delivering drugs to tumor sites and consequently enhancing the chemotherapeutic effects while reducing side effects. Moreover, the strong and stable emission from the CDs on the MSNs means that the nanohybrid fulfills imaging-guided drug delivery by real-time fluorescence tracking. Finally, it is worth noting that the as-prepared nanohybrid showed only superior performance for imaging-guided drug delivery to FR-overexpressing cancer cells (i.e., HeLa cells). Our ongoing work is focusing on using the developed strategy to prepare other MSNs–CDs systems that can target other kinds of cancer cells, with potential applications for cancer treatment in vivo.

## Electronic Supplementary Material

Below is the link to the electronic supplementary material.

Supplementary material 1 (PDF 322 kb)
