# Supplementary material for: In Situ Synthesis of Fluorescent Mesoporous Silica–Carbon Dot Nanohybrids Featuring Folate Receptor-Overexpressing Cancer Cell Targeting and Drug Delivery
Source: Nanomicro Lett. 2019 Apr 5;11:32. doi: 10.1007/s40820-019-0263-3 (PMC7770874; doi:10.1007/s40820-019-0263-3)
Supplement: Supplementary file 1 — Supplementary material 1 (PDF 322 kb) [file 40820_2019_263_MOESM1_ESM.pdf]

Nano-Micro Letters

Supporting Information for

In Situ Synthesis of Fluorescent Mesoporous Silica-Carbon Dot Nanohybrids Featuring Folate Receptor-Overexpressing Cancer Cell Targeting and Drug Delivery

Shuai Zhao[1, 2], Shan Sun[2, *], Kai Jiang[2], Yuhui Wang[2], Yu Liu[3], Song Wu[3], Zhongjun Li[4], Qinghai Shu[1, 3, *], Hengwei Lin[2, *]

[1]School of Material Science and Engineering, Beijing Institute of Technology, Beijing 100081, People's Republic of China

[2]Key Laboratory of Graphene Technologies and Applications of Zhejiang Province, Ningbo Institute of Materials Technology & Engineering, Chinese Academy of Sciences, Ningbo 315201, People's Republic of China

[3]The Affiliated Luohu Hospital of Shenzhen University, Shenzhen Luohu Hospital Group, Shenzhen 518001, People's Republic of China

[4]College of Chemistry and Molecular Engineering, Zhengzhou University, Zhengzhou, 450001, People's Republic of China

*Correspongding authors. E-mail: sunshan@nimte.ac.cn (Shan Sun); qhshu121@bit.edu.cn (Qinghai Shu); linhengwei@nimte.ac.cn (Hengwei Lin)

Supplementary Figures and Table

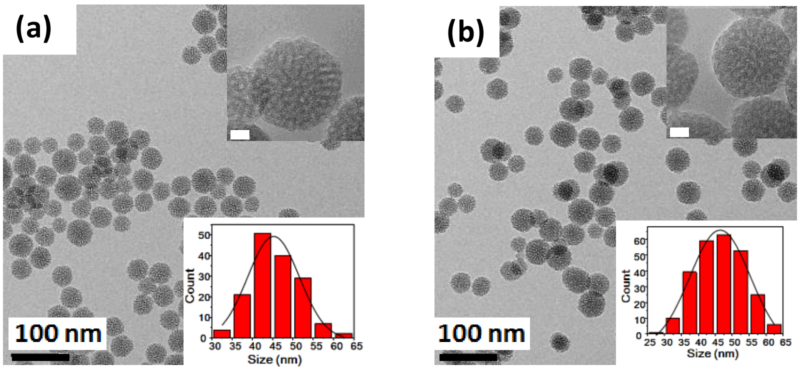

Fig. S1 TEM images of **a** MSNs and **b** MSNs-NH_2. Inset: high resolution images (scale bar: 10 nm) and size distributions

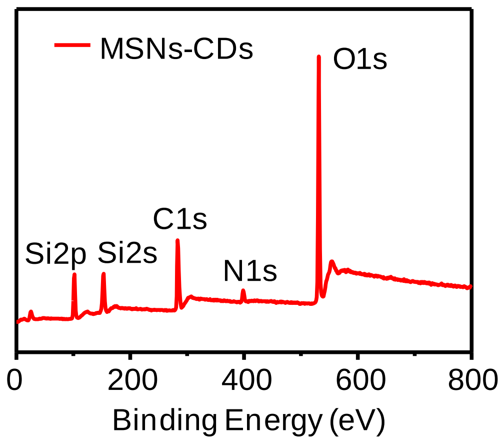

Fig. S2 XPS spectrum of MSNs-CDs

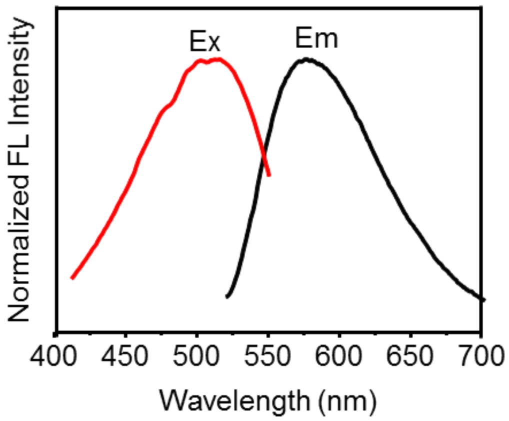

Fig. S3 Fluorescence excitation spectrum (red line, λ_{em}=580 nm) and emission spectrum (black line, λ_{ex}= 510 nm) of MSNs-CDs

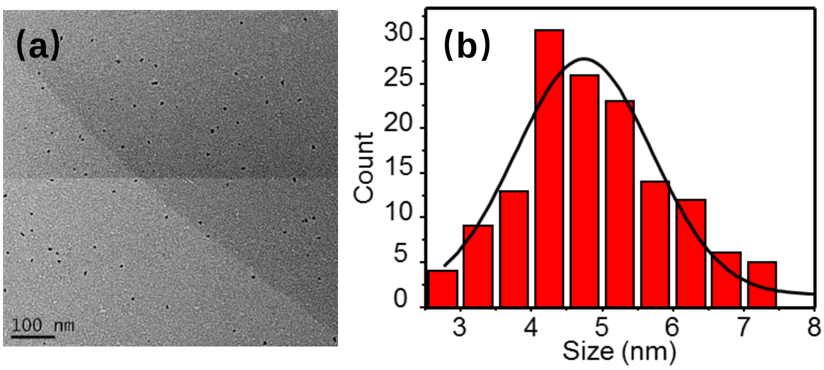

Fig. S4 a TEM image of the CDs harvested from MSNs-CDs with the treatment by HF; **b** Size distribution of the CDs

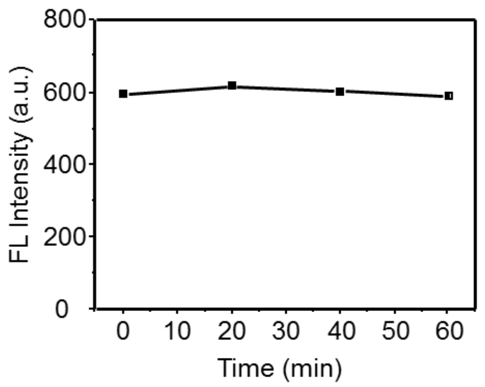

Fig. S5 Photostability of the MSNs-CDs (200 μg mL^{-1}) under continuous irradiation of ultraviolet light

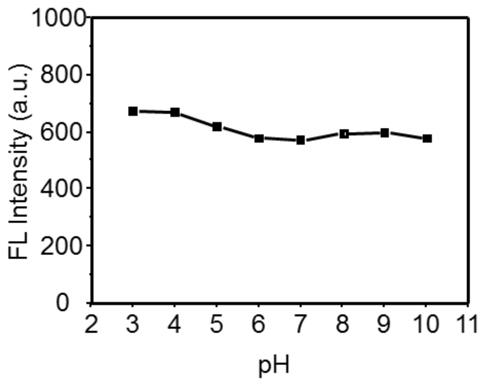

Fig. S6 Fluorescence emission intensities (λ=580 nm) of the MSNs-CDs (200 μg mL^{-1}) at different pH values

Table S1 Parameters of mesoporous structure, including specific surface area, pore size, and pore volume of MSNs, $MSNs\text{-}NH_2$, and MSNs-CDs

Sample	$S_{BET}{}^{a}$ (m^2/g)	$V_t{}^{b}$ (cm^3/g)	$D_{BJH}{}^{c}$ (nm)
MSNs	760.95	1.42	3.12
$MSNs\text{-}NH_2$	554.16	1.22	2.48
MSNs-CDs	502.94	0.88	2.20

$^{a}S_{BET}$: specific surface area based on BET method; $^{b}V_t$: total pore volume at P/P_0 = 0.974 based on BJH analysis; $^{c}D_{BJH}$: averaged pore diameter based on BJH analysis of desorption isotherm.
